# Implementation of Replica-Averaged Restraints from Nuclear Magnetic Resonance Measurement with UNRES Coarse Grained Model of Polypeptide Chains

**DOI:** 10.3390/molecules30224354

**Published:** 2025-11-10

**Authors:** Leonid Shirkov, Cezary Czaplewski, Adam Liwo

**Affiliations:** 1 Faculty of Chemistry, University of Gdańsk, Fahrenheit Union of Universities, Wita Stwosza 63, 80-308 Gdańsk, Poland; leonid.shirkov@umk.pl (L.S.); cezary.czaplewski@ug.edu.pl (C.C.); 2 Institute of Physics, Polish Academy of Sciences, Al. Lotników 32/46, 02-668 Warsaw, Poland

**Keywords:** data-assisted modeling, replica-averaged restraints, UNRES coarse-grained model, nuclear magnetic resonance

## Abstract

We report the implementation of replica-averaged molecular dynamics in the UNRES coarse-grained model of polypeptide chains, with application to the restraints determined by nuclear magnetic resonance. The analytical ESCASA algorithm is used to estimate interproton distances from coarse-grained geometry. With synthetic restraints derived from two selected conformations of the L129–L153 loop of the Slr1183 protein from *Synechocystis* sp. (2KW5), the replica-averaged extension of UNRES retrieved the ensemble of conformations close to the parent structures, with residual content of those not similar to any of them, and comparable populations of both families. Tests with a small putatively multistate protein (PDB: 2LWA) and two proteins with disordered regions (2KW5 and 2KZN, respectively) run in multiplexed temperature replica exchange mode with replica averaging resulted in conformational ensembles that had fewer distance-restraint violations than those deposited in the Protein Data Bank. The ensembles obtained with replica averaging also had fewer distance-restraint violations than those obtained in our previous work, in which time-averaged restraints were implemented. The upgraded UNRES can be used in data-assisted simulations of multistate and intrinsically-disordered proteins and proteins with intrinsically disordered regions.

## 1. Introduction

After AlphaFold was developed and became a routine tool for very reliable modeling of protein structures [1,2], in most cases at the X-ray crystallography resolution, one of the remaining challenges is discerning the conformational ensembles of proteins. Proteins in solution are inherently dynamic [3,4,5] and, consequently, the description of their structures in terms of a single conformation is insufficient. Moreover, many biologically functional proteins are intrinsically disordered (IDPs) or contain intrinsically-disordered regions (IDRs) [6,7,8]. Flexible regions, such as loops, often mediate binding or catalysis [9], while the IDPs and the IDRs play central roles in regulation and signaling and are also implicated in diseases [4,7,8,10,11,12]. Consequently, structural biology has shifted from focusing solely on well-folded proteins to recognizing the functional importance of disorder and conformational heterogeneity.

As opposed to structured proteins, comparative modeling (in a broad sense, including AlphaFold), which makes use of databases of protein structures, cannot currently address the modeling of the flexible structures of IDPs and IDRs, even with the help of artificial intelligence (AI). Physics-based modeling with reliable force fields can, in principle, attempt to tackle the problem [13]. However, the current force fields and methods of conformational search have had only limited success even in modeling the structures of structured proteins. Consequently, the results obtained by using the physics-based force fields must be regarded with caution and always checked against the available experimental data.

Data-assisted modeling, which combines experimental restraints and physics-based force fields, seems to be a plausible way to address the determination of protein ensemble structures [14,15,16]. A broad range of experimental methods, including the nuclear magnetic resonance (NMR) spectroscopy [17,18,19,20], small angle X-ray (SAXS), and neutron (SANS) scattering spectroscopy [21,22], Förster resonance energy transfer (FRET) spectroscopy [22,23], cryo-electron microscopy (cryo-EM) [24], X-ray crystallography [25], and chemical cross-link mass spectroscopy (XL-MS) [26,27,28], can probe different features of structural distributions. The observables provided by these experimental techniques are ensemble averages. It should be noted that the experimental data are often low-resolution (SAXS and SANS [15], FRET [22], XL-MS [28]) or sparse/ambiguous (NMR of flexible proteins [15,29]) and noisy, which makes the problem of ensemble determination challenging [30].

Apart from force-field inaccuracy, one of the problems with physics-based modeling of protein structures is the search for the conformational space. Even with the tremendous advancement of computer hardware [31], all-atom simulations of proteins still require huge supercomputer resources. Coarse-grained models enable us to speed up the simulations by merging groups of atoms into extended interaction sites, thereby reducing the cost of energy and force evaluation and averaging out the fast-moving degrees of freedom [32,33,34,35]. Although coarse graining results in a lower resolution than the all-atom description, coarse-grained models remain sufficiently accurate to capture the conformational behavior of flexible and disordered proteins. All-purpose coarse-grained force fields are statistical [33] or physics-based [34,35], depending on whether the respective interaction potentials are derived by Boltzmann inversion of the distribution and correlation functions calculated from structural databases, usually the Protein Data Bank (PDB) [36], or whether they relate to the all-atom potential energy surfaces. In our laboratory, we have been developing the UNited RESidue (UNRES) physics-based coarse-grained model of polypeptide chains [37,38] which, despite a small number of interaction sites (2 per amino-acid residue), thereby enabling large-scale simulations [39], performs well in unassisted modeling of protein structure and dynamics [38].

NMR is one of the most frequently applied techniques in the determination of protein structures in solution [40,41]. This technique provides the distances between the protons or other paramagnetic nuclei and the information of local conformations in terms of backbone and side-chain dihedral angles, which are derived from chemical shifts and vicinal coupling constants. Because the duration of recording a given signal is comparatively long (of the order of milliseconds), the measured quantities are averaged over time even for proteins which have well-defined structures. Therefore, time-averaged restraints were introduced in NMR-data-assisted simulations long time ago [42,43,44,45]. In this approach, the conformational-dependent quantities are averaged over time, with an exponentially-decaying memory function, during the course of molecular dynamics (MD) simulations. Time averaging has been implemented, e.g., in the AMBER package [46]. In our recent work, we implemented time-averaged molecular dynamics in the UNRES model [47], which enabled us to extend this type of calculations to larger proteins compared to the all-atom implementations. Earlier, we developed the ESCASA algorithm for the calculation of approximate positions of protons from coarse-grained geometry using analytical formulas [48]. The ESCASA algorithm estimates the coordinates of the $H_{i}^{\alpha }$ and $H_{i}^{\beta }$ protons (where *i* is the residue index) from the geometry of the $C_{i-1}^{\alpha }\cdots$ $C_{i}^{\alpha }\cdots$ $C_{i+1}^{\alpha }$ backbone fragment and those the $H_{i}^{N}$ amide proton from the geometry of the $C_{i-1}^{\alpha }\cdots$ $C_{i}^{\alpha }\cdots$ $C_{i+1}^{\alpha }\cdots$ $C_{i+2}^{\alpha }$ fragment. The coordinates of side-chain protons further to $H^{\beta }$ are estimated from those of the respective $C^{\alpha }$ atom and those of the vector pointing from $C^{\alpha }$ to the side-chain center. The respective analytical formulas were parameterized by least-squares fitting of the estimated interproton distances to those calculated from experimental protein structures. Because ESCASA uses analytical formulas for approximate proton coordinates, analytical formulas for the forces due to restraints are available, which enables us to use the algorithm in molecular dynamics. The reader is referred to Ref. [48] for the description of ESCASA. We have demonstrated that our coarse-grained approach with time-averaged restraints produces the conformational ensembles of multistate proteins and proteins with disordered loop regions that result in fewer violations and smaller average violation of NMR-derived distance restraints than the ensembles of structures of flexible proteins deposited in the PDB [47].

Time averaging takes care of the mobility of a single conformation. For small systems that undergo frequent conformational transitions, this approach can be sufficient but, for larger ones, averaging observables over ensembles is desirable if not indispensable. One possibility is ensemble reweighting, in which the statistical weights of the conformers generated without a restraint bias are optimized to reproduce the experimental observables (which are conformational averages) [49,50,51,52]. This approach has been implemented in packages such as Xplor-NIH [51]. However, its efficiency depends on the completeness of the generated ensemble of conformations, which can be problematic for large flexible systems. Replica averaging, on the other hand, which stems from the maximum-entropy principle [53,54,55,56], enables us to include ensemble-averaged restraints at simulation time [55,57,58,59,60]. Conformational sampling (usually by using MD) is run in multiple copies (replicas) and the observables are averaged over the replicas. Although, by the ergodic theorem [61,62], time averaging is equivalent to ensemble averaging, much larger time scales than those covered by simulations are necessary for the assumptions of the ergodic theorem to hold. It seems, therefore, reasonable to try ensemble averaging, in the form of replica averaging, as well. However, because the number of replicas is much smaller than a typical ensemble size, it is not pre-determined which of the two averaging approaches produce more complete ensembles.

In this work, we implemented the replica-averaged restraints in the UNRES model of polypeptide chains to run NMR-data-assisted simulations. We combined the replica-averaging extension of NMR-data-assisted simulations with the multiplexed temperature replica exchange molecular dynamics (MREMD) algorithm [63] already implemented in UNRES [64], which enables us to search the conformational space more efficiently than that at a single temperature. We demonstrate that the replica-average extension of UNRES successfully reconstructs conformational ensembles consistent with synthetic restraints, and, for multistate proteins and proteins with disordered loop regions, yields ensembles that violate fewer NMR-derived restraints than corresponding ensembles deposited in the PDB and those resulting from the time-averaged-restraint approach with UNRES [47].

## 2. Results and Discussion

### 2.1. Stability of Replica-Averaged Simulations

**Figure 1:**
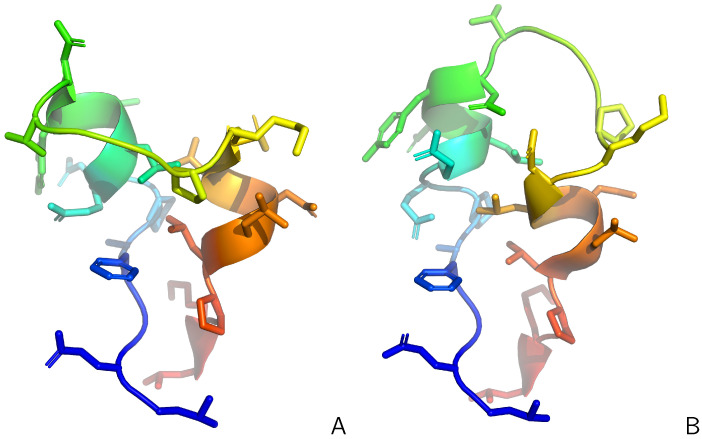
Structures #1 (**A**) and #6 (**B**) of the 2KW5 (L129–L153) used to generate synthetic interproton-distance restraints. The backbones are shown in the cartoon representation and the side chains are shown as sticks, respectively. The chains are colored from blue to red from the N- to the C-terminus. Adapted from Ref. [47]. under the Creative Commons (CC-BY-4.0) license. The pictures were made with PyMOL Version 3.1.6.1 [65].

As in our earlier work on time-average restraints in UNRES [47], introducing replica-averaged restraints with full averaging only every given number of steps and not every step (Section 3.4) causes explicit dependence of the restraint-penalty components of the energy function on time when the average is fully updated [Equation (9)]. Between the full updates, when the averages at a given replica are updated only with the values of the observables from this replica [Equation (11)], the energy function at a given replica does not depend on time explicitly, i.e., it behaves as a potential-energy function. This means that the total (kinetic plus potential) energy in the MD runs carried out in microcanonical mode should exhibit, between the full-update periods, only small oscillations. To determine that this is the case, we used the synthetic restraints derived from structures #1 and #6 of 2KW5 (129–153) (see Figure 1). We carried out a replica-average run in the NVE (constant number of particles, volume, and energy), or microcanonical, mode with 4 replicas for 100,000 MD time steps of the length of Δt=0.489 fs. The starting conformations were randomly generated and subsequently energy-minimized. The initial velocities corresponded to T=300 K (however, the temperature is not conserved in NVE runs). The choice of a small time step was necessary to assess the symplectic behavior of the run between full-replica-averaging steps (the “segment-symplectic” behavior). Full averaging was carried out every $N_{ave}=100$ steps. In this run, the restraint energy was scaled up by the same factor of 4 (the number of replicas) as the forces to enable the assessment of the segment-symplectic behavior of the run.

**Figure 2:**
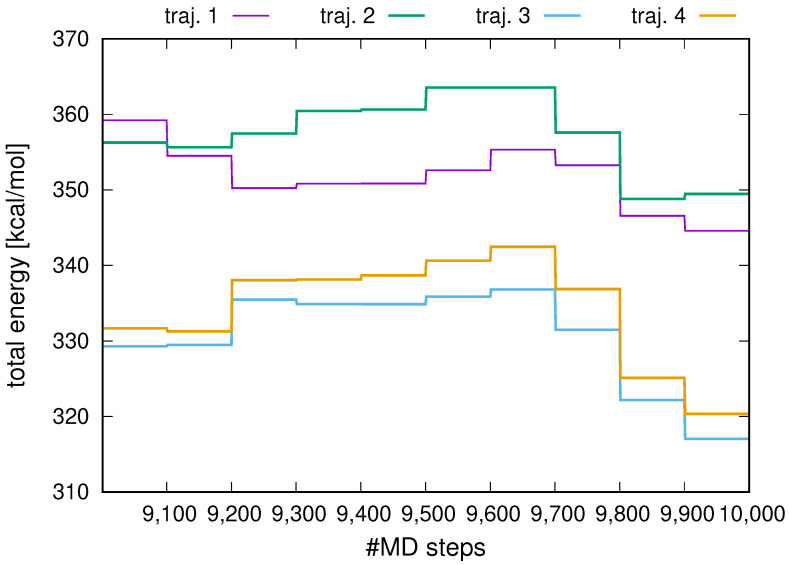
Variation of the total energy with MD step in an microcanonical MD run with UNRES and replica-averaged (4 replicas) distance restraints for 2KW5 (129–153). The plot was made with gnuplot [66].

The plots of the total energy for all 4 replicas, from the 9001-st to the 10,000-th step, are shown in Figure 2. As can be seen, the total energy varies in steps, which correspond to the periods between full replica averaging. Within each step, the total energy is effectively constant, the oscillations being negligible. No energy drift is observed between full replica averaging points. This segment-symplectic behavior of the run enables us to conclude that the replica-averaging algorithm will be stable in canonical and temperature-replica-exchange simulations.

### 2.2. Tests with Synthetic Restraints

To find out how replica averaging affects the structures obtained in restrained simulations, we used the synthetic restraints derived from structures #1 and #6 of 2KW5 (129–153). We carried out replica-averaged simulations with 2 (3 series of 4 runs), 4 (3 series of 2 runs, and 8 (3 runs) replicas, and canonical simulations with no replica averaging (3 series of 8 runs) for reference. Thus, the total number of trajectories was equal to 24 and the number of trajectories in each of the 3 runs or series of runs was 8. All these simulations were carried out in the canonical (NVT; constant number of particles, constant temperature, and constant volume) mode at T=300 K. Each trajectory consisted of 10,000,000 steps at the time step Δt=4.89 fs (a total of 48.9 ns). Snapshots were collected every 10,000 MD steps (1000 snapshots total).

**Figure 3:**
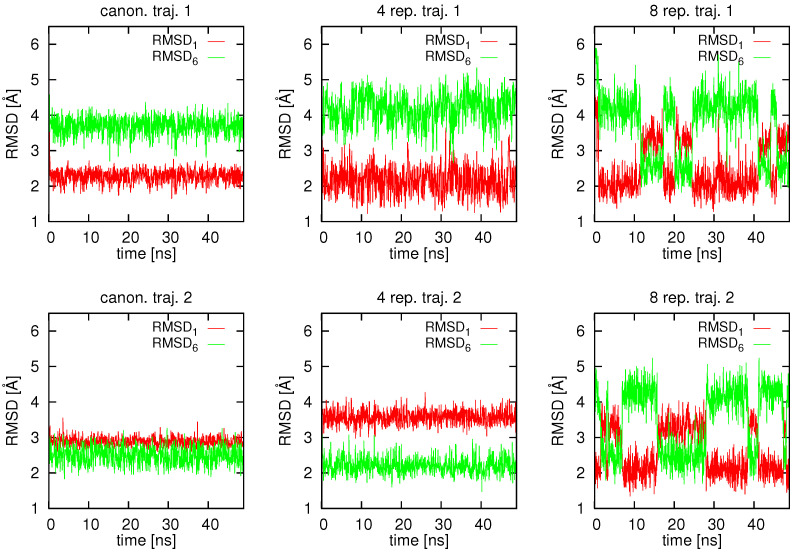
Variation of the RMSD from structure #1 (${\mathrm{RMSD}}_{1}$) and from structure #6 (${\mathrm{RMSD}}_{6}$) in canonical (**left** pair of panels), replica-averaged with 4 replicas (**middle** pair of panels) and replica-averaged with 8 replicas (**right** pair of panels) MD simulations of 2KW5 (129–153) with UNRES and average interproton-distance restraints derived from PDB structures #1 and #6 of 2KW5 (129–153). Two representative trajectories are shown for each calculation mode. The picture was made with gnuplot [66].

In Figure 3, the variation of RMSD from structures #1 (${\mathrm{RMSD}}_{1}$) and #6 (${\mathrm{RMSD}}_{6}$) that were used to derive the average proton-proton distance restraints is shown for 2 representative trajectories of plain canonical simulations (no replica averaging), 2 trajectories taken from a replica-averaged simulation with 4 replicas, and 2 trajectories taken from a simulation with 8 replicas, respectively. As can be seen from Figure 3, one of the two canonical (with no replica averaging) trajectories shown stays around structure #1 and the other one around structure #6, respectively. Thus, running multiple canonical MD trajectories restrained by averaged interproton-distance restraints without any restraint averaging can retrieve both conformations from which the restraints were derived. However, even for this simple system a trajectory is stuck in the initially encountered extended energy basin. This observation was made by earlier researchers who considered model double-well energy functions [53,55,67]. With averaging over 4 replicas, the behavior is qualitatively similar; however, ${\mathrm{RMSD}}_{1}$ and ${\mathrm{RMSD}}_{6}$ vary significantly more and lower values of these quantities are attained. With 8 replicas, quite frequent transitions are observed between structures #1 and #6, which is manifested as reciprocating jumps from low to higher values of ${\mathrm{RMSD}}_{1}$ and ${\mathrm{RMSD}}_{6}$. Such jumps between the two energy basins were also observed in earlier studies in which model double-well potentials were considered [53,55,67].

**Figure 4:**
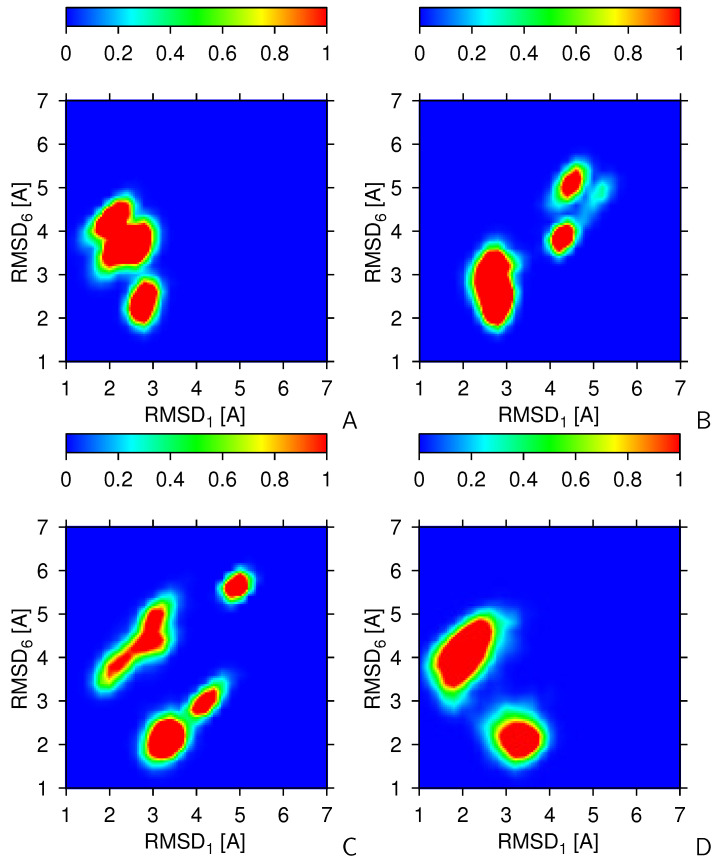
Heat maps maps the $C^{\alpha }$-RMSD distributions from structure #1 (${\mathrm{RMSD}}_{1}$) and #6 (${\mathrm{RMSD}}_{6}$) of 2KW5 (129–153). (**A**) 8 independent canonical trajectories (no replica averaging). (**B**) Another batch of 8 independent canonical trajectories. (**C**) 4 replica-averaged runs, each with 2 replicas. (**D**) 2 replica-averaged runs, each with 4 replicas. The statistics was collected over the second half [5,000,000 time steps of which every 10,000th point (500 snapshots) were collected] of each trajectory. The color scale is above each panel. The plots were made with gri [68].

The above observations translate to the heat maps of the 2-dimensional ${\mathrm{RMSD}}_{1}$ and ${\mathrm{RMSD}}_{6}$ distributions of the simulated conformations shown in Figure 4, which were constructed using the data of the second half (the last 500 snapshots) of two series of 8 canonical runs (panels A and B), one series of 4 2-replica-averaged runs (panel B), and one series of 2 4-replica-averaged runs (panel C). It can be seen that the canonical simulations without replica averaging can happen to visit mainly the regions of structure #1 or structure #6, although the region around structure #1 visited in the simulation series corresponding to Figure 4A consists of 2 lobes. However, as shown in Figure 4B, canonical simulations are also likely to get stuck in regions of the conformational space far from those of structures #1 and #6. Replica averaging over 2 trajectories does not result in a a qualitative improvement regarding the convergence to the regions of the the two parent structures (Figure 4C). With 4 replicas, the obtained conformations are clustered around the parent structures #1 and #6, even though transitions between the regions are still rare (Figure 3). The RMSD distribution maps do not change qualitatively with increasing the number of replicas to 8.

**Figure 5:**
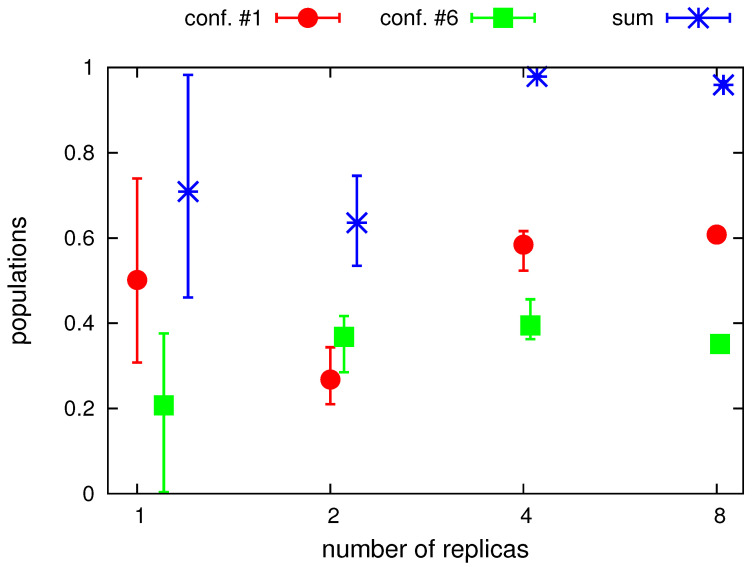
Total populations of conformations assigned to structure #1 and structure #6, respectively, and their sum obtained in canonical (with the “number of replicas” marked as 1 on the abscissa) and replica-averaged simulations with different number of replicas. The symbols are positioned at average values over the three series of each simulation type and the errorbars run from the minimum to the maximum values. The plot was made with gnuplot [66].

In Figure 5, the populations of conformations around structure #1 and around structure #6 and the sum of these populations are plotted in the number of replicas. The conformations were assigned to structure #1, if ${\mathrm{RMSD}}_{1}<{\mathrm{RMSD}}_{6}$ and ${\mathrm{RMSD}}_{1}<3$ Å and to structure #6, if ${\mathrm{RMSD}}_{6}<{\mathrm{RMSD}}_{1}$ and ${\mathrm{RMSD}}_{6}<3$ Å, respectively. As can be seen from the Figure, canonical simulations and the simulations with averaging over 2 replicas leave many conformations outside the regions of structures #1 and #6 (the sum of the populations of the conformations assigned to either of them is remarkably lower than 1). Moreover, the dispersion of the populations from different batches of runs is very high for canonical simulations and still high for the simulations with 2 replicas. With 4 replicas, the number of conformations unassigned to structure #1 or structure #6 is marginal but the dispersion of populations is still noticeable. The dispersion becomes negligible with 8 replicas. Interestingly, based on the results obtained with 4 and 8 replicas, with which asymptotic values of populations seem to have been achieved, it appears that the retrieved populations are about 60% (structure #1) and about 40% (structure #6), respectively, while the synthetic distance restraints computed from the parent structures (Figure 1) were averaged with weights of 0.5. This result is, however, not surprising given the similarity of the two structures and the loss of accuracy inherent in using a heavily coarse-grained model, with which the proton positions are not provided explicitly but are estimated from the coarse-grained geometry by using the ESCASA algorithm [48].

### 2.3. Tests with Experimental Restraints

**Figure 6:**
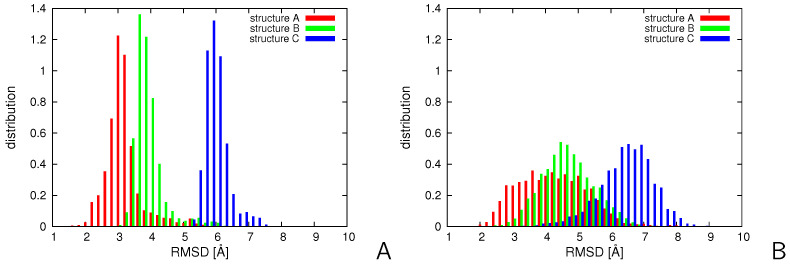
Distributions of $C^{\alpha }$-RMSD of the structures of 2LWA from PDB structures A, B, and C for the conformational ensembles obtained in NMR-data-assisted MREMD simulations (**A**) without replica averaging and (**B**) with replica averaging. The plots were made with gnuplot [66].

We started from the simulations with replica-averaged distance and angular restraints in the temperature replica-exchange mode for the putatively three-state 2LWA small protein [69]. The settings of these calculations are described in Section 3.6. The distributions of the RMSD of the structures of the conformational ensemble at T=280 K from mean structures (chains) A, B, and C, respectively, of the 2LWA PDB entry obtained in simulations with replica-averaged restraints are compared with those obtained with non-averaged restraints in Figure 6. As can be seen, the distributions of the RMSDs from partially-open and open structures B and C obtained with replica-averaged restraints spread to lower RMSD values compared to those obtained with non-averaged restraints. All RMSD distributions become significantly broader and shifted to higher RMSD values following the introduction of replica-averaged restraints. The right shift is strictly connected with broadening because RMSD has the meaning of distance and, consequently, increasing the spread of the distribution of conformations results in the right shift of the mean distance of a conformation from a given reference structure. The results demonstrate that the partially-open and open structures B and C become more visited when the simulations are carried out with replica-averaged restraints. On the other hand, as also found in our earlier study in which time-averaged restraints were applied [47], 2LWA should be considered as an ensemble of conformations gradually passing from the helical hairpin (structure A) to a kinked helix (structure C) rather than as a three-state system.

**Figure 7:**
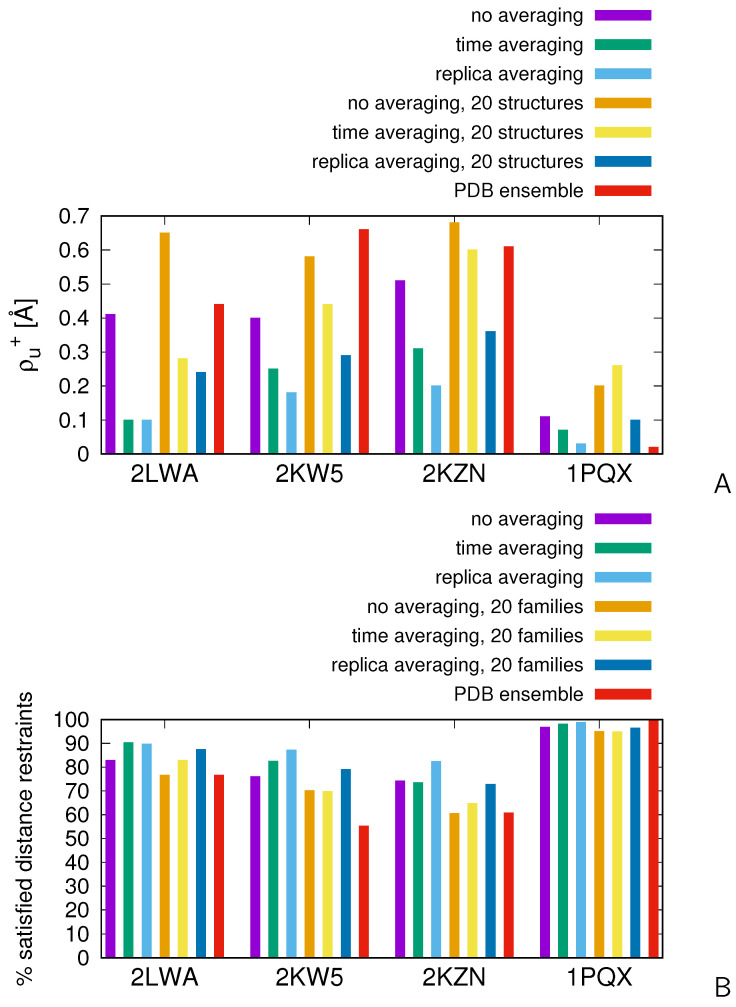
Bar plots of the measures of the compatibility of the ensemble-averaged interproton distances for 2LWA, 1PQX, 2KW5, and 2KZN calculated from the results of MREMD simulations without averaging, with replica-averaged restraints, with time-averaged restraints (the length of the memory window being τ=489 ps and full-average-update frequency being $n_{ave}=1000$ steps, respectively, data from [47]), and those averaged over the structures of the ensembles from the respective PDB entries, respectively. (**A**) Mean-square deviations of the ensemble-averaged interproton distances from the upper boundaries of the NMR-determined distances, $\rho_{u}^{+}$ [Equation (17)]. (**B**) The percentages of satisfied restraints. The errors of $\rho_{u}^{+}$ and those of the percentages of satisfied distance restraints, which were estimated by splitting the set of conformations used in the analysis into two independent subsets and calculating the $\rho_{u}^{+}$ and the percentages of satisfied restraints for each of them, are less than 0.01 Å and less than 1 %, respectively. The plots were made with gnuplot [66].

We subsequently carried out the calculations on the two proteins with disordered loops, 2KW5 and 2KZN, and on 1PQX for reference. In Figure 7, the mean-square deviations from the upper interproton-distance boundaries [$\rho_{u}^{+}$ of Equation (17)] and the percentages of satisfied experimental distance restraints corresponding to restrained simulations without averaging and with replica averaging are shown as bar plots. For comparison, we have also included the results of time-averaged simulations of our previous work [47]. The distances were calculated as averages over the whole ensembles at T=280 K and over 20 structures obtained by cluster analysis. All interproton distances were calculated from the all-atom structures obtained after conversion with cg2all. For reference, the $\rho_{u}^{+}$ values (panel A) and the percentages of satisfied distance restraints (panel B) calculated from the ensembles of the respective PDB entries are shown as red bars. It can be noted that $\rho_{u}^{+}$ is very small and the percentage of satisfied restraints is nearly 100% for 1PQX, which has a well-defined structure. Consequently, assuming that the restraints pertain to a single conformation, which is usually inherent in standard software for NMR structure determination such as CYANA [70], gives good results. Conversely, the $\rho_{u}^{+}$ values are much higher and the percentages of satisfied experimental distance restraints are much lower for the ensembles of the other three proteins deposited in the PDB, which suggests that the structures of these proteins are more diffuse or consist of multiple conformational families and cannot be determined by applying all restraints to a single conformation.

As can be seen from Figure 7, for 2LWA, the mean-square deviation from the experimental distance upper boundaries and the percentage of satisfied distance restraints resulting from replica-averaged simulations are comparable with those resulting from time-averaged simulations when the whole ensembles are considered. Replica averaging results in a somewhat closer agreement with the experimental distances when the averages are calculated from the 20 structures obtained by clustering. The ensembles determined with restrained UNRES in these two calculation modes result in fewer violations and smaller average violation of NMR-derived distance restraints than the ensemble of a total of 60 conformations (structures A, B, and C) of the 2LWA PDB entry.

For the two proteins with disordered regions, 2KW5 and 2KZN, replica averaging calculations give much smaller deviations from the upper distance boundaries and higher percentages of satisfied restraints. For the reference protein, 1PQX, replica averaging also gives a much closer agreement with the experimental data than time averaging. For this protein, the mean-square deviation from the upper distance boundaries and the percentage of satisfied restraints calculated over the whole ensemble resulting from the replica-averaged MREMD simulation at T=280 K are comparable to those obtained by averaging the conformations from the respective PDB entry; however, those corresponding to 20 selected structures exhibit not that good agreement with the experimental restraints. As remarked in our earlier work [47], 1PQX does not have extensive disordered regions and, consequently, the structure determined using standard NMR-data-processing tools at the all-atom level gives structures with a higher resolution than applying a coarse-grained model. It can also be noted that the values of $\rho_{u}^{+}$ are always remarkably lower and the percentages of satisfied restraints are remarkably higher for the ensembles obtained in replica-averaged calculations compared to those obtained in MREMD calculations without time- or replica-averaging.

The ensembles of 20 structures corresponding to the “20 families” sections of Figure 7, the calculated ensemble-averaged interproton distances (for whole ensembles at T=280 K and the sub-ensembles of the representatives of the 20 families obtained by clustering), and the distance boundaries determined by NMR are available as part of the Supplementary Materials.

The numerical values of the measures of the agreement of the ensemble-averaged quantities with the NMR-derived restraints shown in Figure 7 are collected in Table S1 of the Supplementary Materials. The values calculated from the interproton distances estimated from coarse-grained structures using ESCASA [48] for all schemes of replica-averaged calculations are collected in Table S2 of the Supplementary Materials. For comparison, the values obtained without replica averaging but with the same distributions of temperatures and multiplexings are also collected in Table S2. As shown, there seems to be no clear trend as far as the mean-square deviations from the upper distance boundaries ($\rho_{u}^{+}$) are concerned. However, the number of violations is significantly higher with the 24 × 2 scheme. Thus, a minimal number of replicas for which averaging is effective seems to be 4, this observation conforming with the results of detailed analysis of the dependence of the quality of averaging on the number of replicas reported in Section 2.2.

## 3. Methods

### 3.1. UNRES Model of Polypeptide Chains

**Figure 8:**
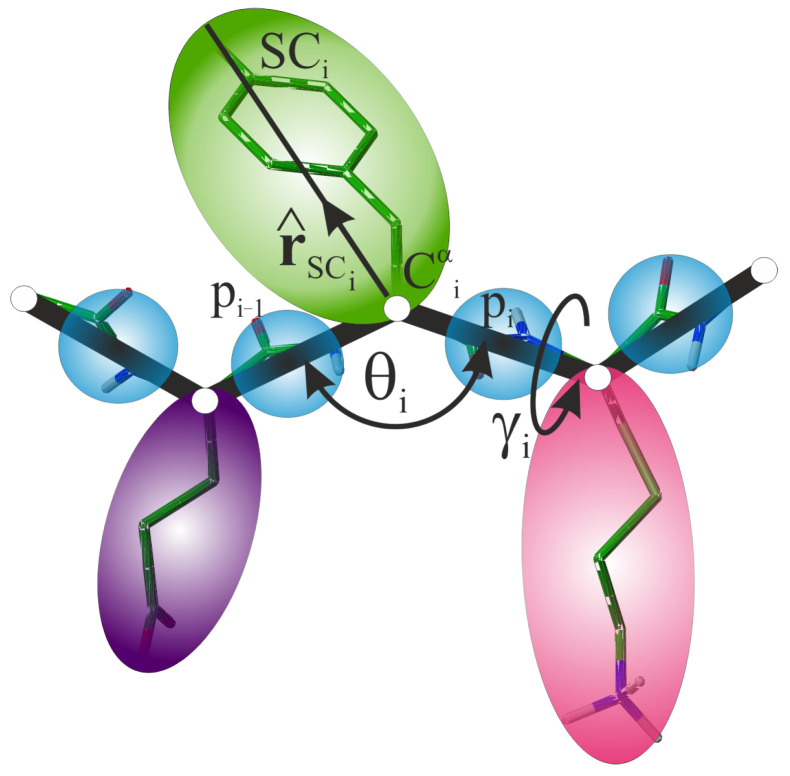
Illustration of the UNRES model of polypeptide chains in the neighborhood of a residue with index *i*. The $C^{\alpha }$ atoms that trace the polypeptide backbone are shown as small white spheres, the peptide groups are shown as blue spheres and the side chains are shown as colored spheroids, respectively. The virtual bonds are shown as lines. The $C^{\alpha }\cdots$ $C^{\alpha }\cdots$ $C^{\alpha }$ virtual-bond angle $\theta_{i}$ and the $C^{\alpha }\cdots$ $C^{\alpha }\cdots$ $C^{\alpha }\cdots$ $C^{\alpha }$ virtual-bond-dihedral angle $\gamma_{i}$ that define local backbone geometry at residue *i*, as well as the unit $C^{\alpha }\cdots$SC vector, ${\hat{r}}_{SC_{i}}$, that defines the orientation of the center of side chain *i* with respect to the respective backbone fragment are marked in the Figure. For illustration, all-atom chain in the stick representation is superposed on the coarse-grained chain. Adapted with permission from Ref. [71]. Copyright 2015 American Chemical Society.

In the UNRES model [37,38], the backbone geometry of a polypeptide chain is defined by the coordinates of the α-carbon ($C^{\alpha }$) atoms, which are connected with virtual bonds. The interaction sites are off the $C^{\alpha }$ atoms; these are the united peptide groups (p), each positioned in the middle between the two consecutive $C^{\alpha }$s and united side chains (SC) attached to the respective $C^{\alpha }$s with virtual bonds (Figure 8). The “side chain” of glycine is located at its $C^{\alpha }$ atom. The Cartesian coordinates of the side-chain centers complete the definition of the geometry of a virtual chain in the UNRES model. The complete Cartesian coordinates of a given system are denoted as the vector q.

The UNRES effective energy function [37,38] is derived from the potential of mean force of polypeptide chains in water [72] and, consequently, depends explicitly on temperature. It consists of site-site interaction, local, and correlation terms. The site-site interaction terms consist of the $U_{SC_{i}SC_{j}}$ potentials for side chain–side chain interactions (these potentials include the solvent-mediated interactions), the $U_{SC_{i}p_{j}}$ potentials for the side chain–peptide group interactions (these are the excluded-volume potentials that control the size of the system), the potentials of the interactions between the peptide groups, which are split into the van der Waals ($U_{p_{j}p_{j}}^{VDW}$) and mean-field electrostatic part ($U_{p_{j}p_{j}}^{el}$), the latter accounting for backbone hydrogen bonding out of the context of local conformational states, and the disulfide bonds, $U_{i,j}^{ssbond}$, potential that accounts for the formation and breaking of disulfide bonds. The local terms consist of the virtual-bond potentials $U_{bond}\left(d_{i}\right)$, where $d_{i}$ is the length of the *i*th virtual bond, the virtual-bond-angle potentials, $U_{b}\left(\theta_{i}\right)$, the virtual-bond-torsional potentials, $U_{tor}\left(\gamma_{i},\theta_{i},\theta_{i+1}\right)$, and the side-chain-rotamer potentials, $U_{rot}\left(\theta_{i},{\hat{r}}_{SC_{i}}\right)$. The correlation terms consist of the $U_{corr}^{\left(3\right)}$ potentials that describe the coupling of the local interactions with backbone-electrostatic interactions between the peptide groups that are far in the amino-acid sequence and the $U_{turn}^{\left(3\right)}$ potentials pertaining to the peptide groups that are second neighbors in the sequence [72]; these terms are essential to reproduce regular secondary structures. The solvent is implicit in UNRES and the solvent-mediated interactions are mainly present in the $U_{SC_{i}SC_{j}}$ potentials. All energy terms are combined with weights (which are determined by force-field calibration) and multiplied by appropriate temperature factors to reflect the dependence of the potential of mean force on temperature [73], as given by Equation (1).

(1) U=wSCSC∑i<jUSCISCJ+wSCp∑i≠jUSCipj+wppVDW∑i<j−1UpipjVDW=+f2(T)wppel∑i<j−1Upipjel+wssbond∑Cyspairsi,jUi,jssbond+wbond∑iUbond(di)=+wb∑iUb(θi)+wrot∑iUrot(θi,r^SCi)+f2(T)wtor∑iUtor(γi,θi,θi+1)=+f3(T)wcorr(3)∑i<j−2Ucorr;pIpJ(3)+f3(T)wturn(3)∑iUturn;pipi+2(3)

with

(2) $$f_{n}\left(T\right)=\frac{\ln[\exp(1)+\exp(-1)]}{\ln\left\{\exp\left[{\left(\frac{T}{T_{\circ }}\right)}^{n-1}\right]+\exp\left[-{\left(\frac{T}{T_{\circ }}\right)}^{n-1}\right]\right\}}$$

The $U_{SC_{i}SC_{j}}$ and $U_{p_{j}p_{j}}^{el}$ potentials have the axial and not the spherical symmetry. This feature and the presence of the correlation terms contribute to the ability of UNRES to model protein structures and dynamics at a good accuracy level despite aggressive coarse graining [38]. A detailed description of the energy function and its derivation can be found in our earlier work [37,38,72].

In the present work, we used the NEWCT-9P variant of UNRES, which was calibrated using a maximum-likelihood approach with a set of nine proteins representing diverse structural classes [37].

### 3.2. Conformational Search with UNRES

The main conformational-search engine of UNRES is Langevin molecular dynamics, which was implemented in our earlier work [74,75]. The respective computer code was subsequently parallelized with Message Passing Interface (MPI) libraries and optimized for speed and memory with parallelization extended to the hybrid Open-MP/MPI mode [39]. A stochastic variant of the velocity Verlet algorithm [76] was developed [75].

To improve sampling efficiency, MREMD was implemented in UNRES [64], including the consideration of the the temperature dependence of the UNRES energy function [73]. MREMD is an extended variant of replica exchange molecular dynamics (REMD), also termed parallel tempering [77,78]. In REMD, multiple trajectories (replicas) are run, each at a different temperature. Each $N_{exch}$ steps, the temperatures are exchanged between replicas based on the Boltzmann criterion. Following this exchange, a trajectory run at a low temperature, in which the system is stuck in a locally high-energy basin, gets a higher temperature to enable the system to leave the kinetic trap. Conversely, a trajectory run at a high temperature that produced low-energy structures gets a lower temperature, this enabling the system to explore the respective low-energy region of the conformational space in more detail. In MREMD, multiple trajectories are run at each temperature, which enables a system to explore the conformational space even more efficiently [63].

Using REMD and MREMD implies the necessity of translating the resulting multicanonical ensemble that consists of structures simulated at multiple temperature to canonical ensemble(s) corresponding to given temperature(s). For this purpose, we adapted [73] the binless variant of the weighted histogram analysis method (WHAM) [79] to the UNRES temperature-dependent energy function.

### 3.3. Restraints from NMR with UNRES

As in our earlier work [47], the restraints derived from NMR experiments are introduced as penalty terms added to the UNRES energy function [Equation (1)]. In the present study, we imposed restraints on (i) the distances between the protons of different residues, (ii) the $C^{\alpha }\cdots$ $C^{\alpha }\cdots$ $C^{\alpha }$ virtual-bond angles (θ), and (iii) the $C^{\alpha }\cdots$ $C^{\alpha }\cdots$ $C^{\alpha }\cdots$ $C^{\alpha }$ virtual-bond dihedral angles (γ) shown in Figure 8.

Proton coordinates required for the computation of the interproton distances are estimated analytically from the coarse-grained geometry using the ESCASA algorithm [48]. The restraints on the θ and γ angles are derived from the boundaries of the backbone ϕ and ψ dihedral angles determined by NMR following Ref. [80]. The extended energy function that includes the restraint terms is given by Equation (3).

(3) $$U=U_{\mathrm{UNRES}}+V_{\mathrm{dist}}+V_{\theta }+V_{\gamma }$$

where $U_{\mathrm{UNRES}}$ is the UNRES energy, $V_{\mathrm{dist}}$ is the distance-restraint penalty term, and $V_{\theta }$ and $V_{\gamma }$ are the angular penalty terms, respectively. The penalty terms are expressed by Equations (4)–(6) [81].

(4) $$V_{\mathrm{dist}}\left(d,d_{l},d_{u},A\right)=\left\{\begin{array}{cc} A\frac{{\left(d-d_{l}\right)}^{4}}{\sigma^{4}+{\left(d-d_{l}\right)}^{4}}\left[1+\kappa \ln\cosh(d-d_{l})\right], & d<d_{l},\\ 0, & d_{l}\leq d\leq d_{u},\\ A\frac{{\left(d-d_{u}\right)}^{4}}{\sigma^{4}+{\left(d-d_{u}\right)}^{4}}\left[1+\kappa \ln\cosh(d-d_{u})\right], & d>d_{u}, \end{array}\right.$$

where *d* is the proton–proton distance (estimated from coarse-grained coordinates), $d_{l}$ and $d_{u}$ are the lower and the upper boundary, respectively, determined by NMR, σ is the effective thickness of the restraint-well wall, *A* is the well depth, and κ is the asymptotic slope of the penalty-term well [81]. In this work, we set A=5 kcal/mol, σ=1 Å, and κ=0.01 in all calculations.

The angular penalty terms are defined be Equations (5) and (6), respectively.

(5) $$\begin{array}{ccc} V_{\theta } & = & A_{\theta }\;g\left(\theta ,\theta_{l},\theta_{u}\right) \end{array}$$

(6) $$\begin{array}{ccc} V_{\gamma } & = & A_{\gamma }\;g\left(\gamma ,\gamma_{l},\gamma_{u}\right) \end{array}$$

with

(7) $$\begin{array}{ccc} g(x,x_{l},x_{u}) & = & \left\{\begin{array}{cc} \frac{1}{4}\delta^{4}, & \delta <\frac{x_{l}-x_{u}}{2},\\ 0, & \frac{x_{l}-x_{u}}{2}\leq \delta \leq \frac{x_{u}-x_{l}}{2},\\ \frac{1}{4}\delta^{4}, & \delta >\frac{x_{u}-x_{l}}{2}, \end{array}\right. \end{array}$$

(8) $$\begin{array}{ccc} \delta & = & \left(x-\frac{x_{l}+x_{u}}{2}\right)\;\mathrm{mod}\;2\pi . \end{array}$$

Here, $\theta_{l}$, $\theta_{u}$, $\gamma_{l}$, and $\gamma_{u}$ are the restraint boundaries on θ and γ. We set $A_{\theta }=1$ kcal/mol and $A_{\gamma }=5$ kcal/mol, respectively.

### 3.4. Replica-Averaged Restraints

To obtain replica-averaged restraints, we average the conformation-dependent quantities that appear in the penalty functions defined by Equations (4)–(6) over the trajectories (replicas) that are, at the moment, run at the same temperature. Let us consider a simulation consisting of *M* replicas run at a single bath temperature first, i.e., with no temperature replica exchange. The replica-averaged quantity ${\bar{y}}_{i}\left(t_{k}\right)$ at time step $t_{k}$ is defined by Equation (9).

(9) $${\bar{y}}_{i}\left(t_{k}\right)={\left[\frac{1}{M}\sum_{j=1}^{M}{\left(y_{ji}\left(t_{k}\right)\right)}^{-m}\right]}^{-\frac{1}{m}},\;i=1,2,\cdots ,N_{y}$$

where *j* is replica index, *i* is the observable index, $N_{y}$ is the number of observables of kind *y*, and *m* is the exponent in averaging. For distances, m=3 and, for angle-dependent quantities, m=−1.

Equation (9) applies as it is to interproton distances and to the virtual-bond angles θ; however, to get the average values of the virtual-bond dihedral angles γ, cosγ and sinγ are replica-averaged and the average value of γ is obtained via the atan2 function, as given by Equation (10).

(10) $$\bar{\gamma }\left(t_{k}\right)=\mathrm{atan}2\left(\bar{\sin\gamma }\left(t_{k}\right),\bar{\cos\gamma }\left(t_{k}\right)\right)$$

where atan2(y,x) is equal to arctan(y/x) in absolute value, while its sign is determined by those of *y* and *x*, which are proportional to the sine and to the cosine of the resulting angle, respectively.

With averaging applied every MD step, a given replica-averaged simulation with *M* replicas can be considered as a single extended MD simulation with the potential energy being the sum of the extended energies [Equation (3)] over all *M* replicas. With the simulation run in the microcanonical mode, the total energy summed over all replicas should be conserved during such an extended MD run. However, taking the averages every time step causes excessive communication between the processes running the respective trajectories, which impairs performance, particularly when there are many restraints and replicas. Moreover, when replica averaging is combined with temperature replica exchange, the averaging changes at replica-exchange points, making the extended energy function explicitly dependent on time at these point. Therefore, we calculate the averages as given by Equation (10) only every $N_{ave}$ number of MD steps; in this work we set $N_{ave}=100$. This modification results in losing extended total energy conservation in the microcanonical mode but, on the other hand, the total energy is conserved for each trajectory between the full-averaging points, as shown in Section 2.1. Between the full-averaging points, we update the average at replica with index *J* with the value of $y_{i}\left(t_{k}\right)$ at time step $t_{k}$ calculated at replica *J*, as given by Equation (11).

(11) y¯i(J)(tk)=1M∑j=1j≠JMyji(tK)−m+yJi(tk)−m−1m,J=1,2,⋯,M,i=1,2,⋯,Ny

where $K=N_{ave}\left(k÷N_{ave}\right)$ is the index of the previous time step at which full update of the average following Equation (9) took place. The averages are calculated starting from $N_{ave}$th time step; until then non-averaged observables are used to compute the values of the penalty functions and gradients.

The gradients of ${\bar{y}}_{i}\left(t_{k}\right)$ at the replica with index *J* is computed from Equation (12).

(12) $$\nabla_{q}{\bar{y}}_{i}^{\left(J\right)}\left(t_{k}\right)=\frac{1}{M}{\left(\frac{y_{Ji}\left(t_{K}\right)}{{\bar{y}}_{i}^{\left(J\right)}\left(t_{k}\right)}\right)}^{m+1}\nabla_{q}y_{Ji}\left(t_{k}\right)$$

where q denotes the vector of UNRES coordinates.

It should be noted that the gradients of the penalty functions can become small with large *M*, a problem that appeared in our previous work, in which we introduced time-averaged restraints into UNRES [47]. Consequently, we scale up the gradients of the penalty functions [Equations (4)–(6)] by *M*, as given by Equation (13).

(13) $$\nabla_{q}V_{y}\leftarrow M\nabla_{q}V_{y}$$

where *y* denotes the type of the restraint.

When the replicas are run at multiple temperatures, the averaging should, in principle, be carried out over all replicas with appropriate weights, which vary depending on the temperature of the replica under consideration, as given by Equation (14). We assume that the number of multiplexings, *M*, is independent of temperature.

(14) $$\begin{array}{ccc} {\bar{y}}_{Ti}\left(t_{k}\right) & = & {\left(\sum_{j=1}^{N_{T}M}w_{jT}y_{ji}{\left(t_{k}\right)}^{-m}\right)}^{-\frac{1}{m}} \end{array}$$

(15) $$\begin{array}{ccc} \sum_{j=1}^{N_{T}M}w_{jT} & = & 1 \end{array}$$

where ${\bar{y}}_{Ti}\left(t_{k}\right)$ is the *i*th observable average of kind *y* at replica temperature *T*, $y_{ji}\left(t_{k}\right)$ is the value of the observable for the *j*th replica, $w_{jT}$ is the weight of the replica with index *j* at temperature *T* (which is not necessarily the temperature at which the replica is run at the moment).

In the present work, we assumed that only the replicas run at the temperature *T* at full-averaging time contribute to the averages computed at that temperature. Consequently, the weights are expressed by Equation (16); with this definition the weights are normalized to 1 [see Equation (15)].

(16) $$w_{jT}=\left\{\begin{array}{cc} \frac{1}{M} & \mathrm{if}\;\mathrm{replica}\;j\;\mathrm{is}\;\mathrm{run}\;\mathrm{at}\;\mathrm{temperature}\;T,\\ 0 & \mathrm{otherwise}. \end{array}\right.$$

With the above definition of weights and given the same multiplexing *M* of replicas per each temperature, the forces are still to be scaled up as given by Equation (13).

Clearly, the frequency of full updates of replica averages, $N_{ave}$, must be a sub-multiple of the frequency of exchanging temperature replicas, $N_{exch}$ In this work, we apply $N_{exch}=10,000$ and, thus, with $N_{ave}=100$, this condition is fulfilled.

Replica averaging and temperature replica exchange are run in the parallel mode. In this work, MPI routines were used to construct the replica-average part of the UNRES code.

### 3.5. Systems Studied and Restraints

**Figure 9:**
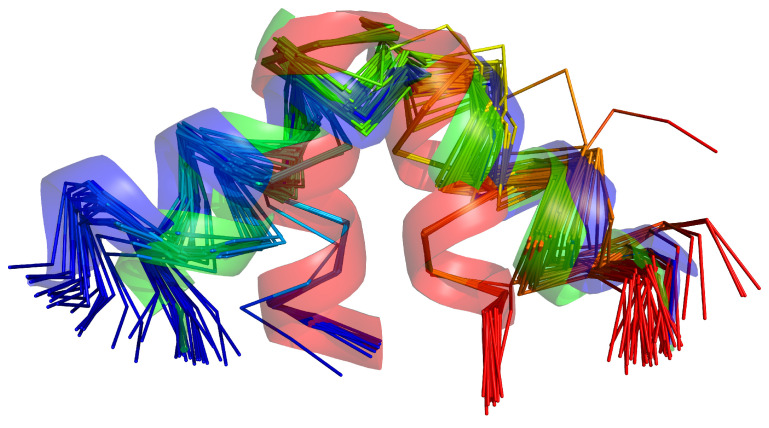
NMR ensemble of the 2LWA forms consisting of structures A, B, and C of the respective PDB entry. The first conformation of each family of structures is shown as a transparent cartoon, colored red for structure A, green for structure B, and blue for structure C, respectively. The other conformations of each family are shown in $C^{\alpha }$-trace representation, chains colored from blue to red from the N- to the C-terminus. The picture was made with PyMOL [65].

**Figure 10:**
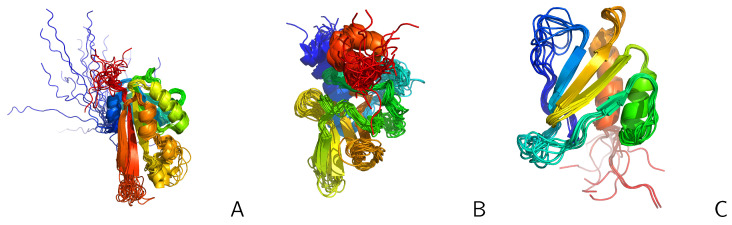
NMR ensembles of the three proteins from the Montelione/NEF Benchmark Set [83]: (**A**) 2KW5, (**B**) 2KZN, (**C**) 1PQX in the cartoon representation. The chains colored blue to red from N- to C-terminus. The disordered loops are exposed. The pictures were made with PyMOL [65].

We tested UNRES with replica-averaged restraints with the same systems as in our earlier work on time-averaged restraints [47], which enabled us to compare the behavior of replica-averaged restraints with that of time-averaged restraints: the L129–L153 loop of the Slr1183 protein from *Synechocystis* sp. (PDB: 2KW5 [82]), hereafter referred to as 2KW5 (129–153), the influenza hemagglutinin fusion peptide (PDB: 2LWA [69]), which is a small, putatively three-state protein (PDB: 2LWA), whose NMR ensemble includes three families of conformations, referred to as chains A, B, and C, respectively, in the PDB entry, which we hereafter refer to as structures A, B, and C, respectively, and three proteins from the Montelione/NEF Benchmark Data Set [83], namely the full-length 2KW5, the peptide methionine sulfoxide reductase msrB from *Bacillus subtilis* (PDB: 2KZN), hereafter referred to as 2KZN, which have large disordered loops, and the *Staphylococcus aureus* protein SAV1430 (PDB: 1PQX), hereafter referred to as 1PQX, which has a much tighter structure and is modeled well without restraint averaging [47]. 2KW5 (129–153) was run with synthetic restraints averaged over two conformations, referred to as structure #1 and structure #6, respectively, which are shown in Figure 1. The superposed families of NMR structures of 2LWA are shown in Figure 9 and the NMR structures of 2KW5, 2KZN, and 1PQX are shown in Figure 10, respectively.

For 2KW5, we used the set of 186 synthetic distance restraints generated in our previous work [47], which were computed as the inverse sixth power averages over the two conformations shown in Figure 1 with equal weights. The distance and angular restraints of the other systems were taken from the respective PDB entries; we used the same refined restraints labeled “v2”, as in our previous work [47]. The purpose of using synthetic restraints from only two conformations was to assess the behavior of replica averaging in a clear way, as it was done in our earlier work on introducing the time-averaged extension into UNRES [47]. Although it could be expected that two families, each containing conformations similar to either the first or the second of the parent conformations will be obtained, a possibility could not be excluded that the method would produce an ensemble with conformations that are not similar to any of the parent conformations, while the ensemble would satisfy the synthetic distance restraints. The numbers of experimental restraints for each protein are summarized in Table 1. All restraints converted to the format read by UNRES are available as Supporting Information to Ref. [47].

### 3.6. Calculation Procedure

For all systems except 2KW5 (L129–L153), we carried out restrained MREMD simulations, with replica-averaged restraints and non-averaged restraints for reference. We used 4 sets of replicas, which are labeled with $N_{T}\times M$, where $N_{T}$ is the number of temperatures and *M* is the number of multiplexings (same for each temperature), each consisting of a total of 48 trajectories ($N_{T}\times M=48$), as shown in Table 2. As per Equation (16), the number of multiplexings also is the number of replicas over which the averaging is performed.

The bulk of the analysis was carried out for the 12×4 setting, which was also used in our earlier work on UNRES with time-averaged restraints [47].

Each trajectory consisted of a total of 20,000,000 time steps with the 4.89 fs step length. Replicas were exchanged and snapshots were collected every 10,000 steps and, in the replica-averaged calculations, the full averages were computed every 100 steps. The calculations were carried out in the Langevin-dynamics mode, with the water friction scaled down by a factor of 0.05, as in our earlier work [47] and the variable time step (VTS) algorithm [74] was used to integrate the equations of motion. The starting conformations were randomly generated, using the algorithm described in our earlier work that builds a polypeptide chain in the UNRES representation subject to the non-overlap condition [84], and subsequently energy minimized. For each trajectory, a different random starting conformation was generated.

After an MREMD run was finished, binless WHAM was executed using the conformations of the second half of each trajectory (1000 conformations per trajectory), with the sampling frequency of 8, this yielding 6000 conformations. Subsequently, the weights of the conformations were computed at T=280 K, as in our earlier work [47], and the conformations were converted to the all-atom representation by using the cg2all algorithm from Feig’s lab [85,86]. The interproton distances were calculated for each conformation and their inverse sixth power weighted averages at T=280 K were computed, as described in our earlier work [47] and compared with the values determined by NMR. For comparison, we also considered the average interproton distances estimated by ESCASA [48]. As in our earlier work [47], we used two measures of fitting the calculated to the experimental restraints. The first one was the root mean square deviation of the ensemble-averaged interproton distances from the upper distance boundaries ($\rho_{u}^{+}$), defined by Equation (17), the second one was the percentage of distances within the distance boundaries (effectively smaller than the respective upper boundaries). Additionally, we considered the number of upper-boundary violations ($n_{viol}$; the number of distances greater than the upper distance boundaries) and the number of gross upper-boundary violations ($N_{viol}$; the number of distances greater by 2 Å or more than the upper distance boundaries).

(17) $$\begin{array}{ccc} \rho_{u}^{+} & = & \sqrt{\frac{1}{N_{d}}\sum_{i=1}^{N_{d}}\delta_{i}^{2}} \end{array}$$

(18) $$\begin{array}{ccc} \delta_{i} & = & \left\{\begin{array}{cc} {\bar{d}}_{i}-d_{i}^{u} & d_{i}>d_{i}^{u}\\ 0 & \mathrm{otherwise} \end{array}\right. \end{array}$$

where $N_{d}$ is the number of interproton-distance restraints, ${\bar{d}}_{i}$ is the ensemble-averaged distance, and $d_{i}^{u}$ is the upper distance boundary.

The number of conformations with significant weights that contributed to averaging was typically greater than 1000, which is much greater than 10–60 conformations of an NMR-determined protein structure deposited in the PDB. Therefore, as in our earlier work [47], we applied Ward’s minimum variance clustering [87] to dissect each set into 20 families. From each family, we selected the conformation best satisfying the experimental NMR restraints, computed the weighted inverse sixth power averages (the weights being the cumulative statistical weights of all conformations of the respective families) of the interproton distances and compared them with the respective NMR-determined values.

To compare the quality of the ensembles determined by NMR-restrained UNRES simulations with the PDB ensembles, we used the sixth-power averages of the distances calculated from the respective PDB ensembles in our earlier work [47].

The specific settings for the calculations with synthetic restraints, in which no temperature replica exchange was involved, and which were aimed at testing the stability of the replica-average algorithm and the dependence of results on the number of replicas are described in Section 2.1 and Section 2.2, respectively.

## 4. Conclusions

In this work, we implemented replica-averaged restraints in the UNRES package for coarse-grained simulations of proteins to enable NMR-data-assisted simulations of the structures of flexible proteins. With a model system, for which interproton distance restraints were derived from two conformations, we have demonstrated that replica averaging prevents the system from getting stuck in local extended-energy basins, which can be far from the neighborhood of the parent conformations. Similar observations were made in earlier work in which model systems were considered [53,55,67]. The facilitated walking in the conformational space translates to generating conformational ensembles with fewer violations and smaller average violation of NMR-derived distance restraints than those obtained without replica averaging. For the putatively multistate protein (2LWA) and proteins with disordered regions (2KW5 and 2KZN), the obtained ensembles, even reduced to the customary size of 20 structures, satisfied more experimental restraints than those deposited in the PDB (Figure 7), although the heavily coarse grained UNRES model was used in conformational search, which inevitably impaired the resolution of the obtained models. An important practical conclusion from our study is that a modest number of replicas (4 per temperature) is sufficient to obtain good averaging. Consequently, the computing time is comparable with that of the standard MREMD-based protocol of protein-structure modeling with UNRES [38].

The replica-averaging approach developed in this work can be applied directly to other types of data-assisted simulations that provide ensemble-averaged observables such as FRET. It can easily be extended to handle SAXS/SANS data, in which the distance distribution [88] or, directly, the scattering intensity is restrained [89,90]. Extension is also possible to XL-MS data, which yield cross-linked fragments apparently coming from the protein molecules which are momentarily in conformations that are not compatible with each other, a feature causing difficulties in data-assisted modeling [91]. These extensions are currently being developed in our laboratory.

Compared to time-averaged restraints implemented in UNRES in our earlier work [47], using replica-averaged restraints appears to result in the conformational ensembles of improved compatibility with the experimental data. This result is understandable because time averaging pertains to the same trajectory at all times. Consequently, with growing size of the system under study, the averaging is carried out over a smaller set of conformations. Conversely, averaging over replicas, which explore different regions of the conformational space, is more extensive. Moreover, when averaging is done in the temperature replica-exchange mode, the replicas to average over can potentially change after the replica-exchange points [Equations (14)–(16)], thus making the averaging even more extensive. On the other hand, NMR measurements result in observables that are averaged both over time and conformational ensembles. Therefore, the NMR-data-assisted approach to protein simulations should combine time- and replica-averaging, which would reflect local conformational diversity during the time a signal is collected and global diversity resulting from collecting signals from the whole ensemble. This research is currently being carried out in our laboratory.

## Figures and Tables

**Table 1:** Numbers of residues and NMR-derived restraints on interproton distances (*d*), backbone virtual-bond dihedral angles γ, and backbone virtual-bond angles θ (the angles are illustrated in Figure 8) used in UNRES/MREMD simulations.

Protein	# res.	Restraint Type		
		d	γ	θ
2LWA	25	175	18	20
2KW5	202	947	134	147
2KZN	147	578	104	121
1PQX	91	1015	64	74

**Table 2:** The sets of replica temperatures and multiplexings used in calculations.

Set	Temperatures [K]											
24×2	262	264	267	270	274	277	279	282	285	288	290	292
	295	298	301	305	308	315	327	333	340	355	362	370
12×4	262	267	274	279	285	290	295	301	308	333	355	370
8×6	274	279	285	290	295	301	308	333				
6×8	279	285	290	295	301	308						

## Data Availability

The FORTRAN code of the algorithm together with the examples presented in this paper are available at https://unres.pl/downloads under the “Time- and replica average enabled UNRES” entry, URL accessed at 7 November 2025. The simulated structures and ensemble-averaged distances are available from Supplementary Materials.

## Supplementary Materials

The following supporting information can be downloaded at: https://www.mdpi.com/article/10.3390/molecules30224354/s1, Table S1: Right RMSDs from the upper distance boundaries ($\rho_{u}^{+}$s), the total numbers of violated restraints ($n_{viol}$), and the numbers of restraints violated by more than 2 Å ($N_{viol}$) for the 2LWA, 1PQX, 2KW5, and 2KZN proteins from MREMD simulations with 12 quadruplexed replicas; Table S2: Right RMSDs from the upper distance boundaries ($\rho_{u}^{+}$s), the total numbers of violated restraints ($n_{viol}$), and the numbers of restraints violated by more than 2 Å ($N_{viol}$) for the 2LWA, 1PQX, 2KW5, and 2KZN proteins calculated from the ESCASA-estimated proton coordinates from four schemes of NMR-data-restrained MREMD simulations with UNRES in the replica-average mode and without averaging; ASCII files: Simulated structures of the benchmark proteins and (files 2LWA_20_12x4.pdb, 2KW5_20_12x4.pdb, 2KZN_20_12x4.pdb, and 1PQX_20_12x4.pdb); ASCII files: Experimental and calculated upper distance boundaries averaged over whole simulated ensembles at T=280 K (files 2LWA_280_allat_aveall.nmr, 2KW5_280_allat_aveall.nmr, and 2KZN_280_allat_aveall.nmr, 1PQX_280_allat_aveall.nmr) and over 20 structures selected by cluster analysis (files 2LWA_280K_20models_aveall.nmr, 2KW5_280K_20models_aveall.nmr, 2KZN_280K_ 20models_aveall.nmr, and 1PQX_280K_20models_aveall.nmr).

## References

[1] Senior A.W., Evans R., Jumper J., Kirkpatrick J., Sifre L., Green T., Qin C., Žídek A., Nelson A.W.R., Bridgland A. (2020). Improved protein structure prediction using potentials from deep learning. Nature.

[2] Jumper J., Evans R., Pritzel A., Green T., Figurnov M., Ronneberger O., Tunyasuvunakool K., Bates R., Žídek A., Potapenko A. (2021). Highly accurate protein structure prediction with AlphaFold. Nature.

[3] McCammon J.A., Gelin B.R., Karplus M. (1977). Dynamics of folded proteins. Nature.

[4] Henzler-Wildman K., Kern D. (2007). Dynamic personalities of proteins. Nature.

[5] Vendruscolo M., Dobson C.M. (2011). Protein dynamics: Moore’s law in molecular biology. Curr. Biol..

[6] Dunker A.K., Lawson J.D., Brown C.J., Williams R.M., Romero P., Oh J.S., Oldfield C.J., Campen A.M., Ratliff C.M., Hipps K.W. (2001). Intrinsically disordered protein. J. Mol. Graph. Model..

[7] van der Lee R., Buljan M., Lang B., Weatheritt R.J., Daughdrill G.W., Dunker A.K., Fuxreiter M., Gough J., Gsponer J., Jones D.T. (2014). Classification of intrinsically disordered regions and proteins. Chem. Rev..

[8] Uversky V.N. (2014). Intrinsically Disordered Proteins.

[9] Amaral M., Kokh D.B., Bomke J., Wegener A., Buchstaller H.P., Eggenweiler H.M., Matias P., Sirrenberg C., Wade R.C., Frech M. (2017). Protein conformational flexibility modulates kinetics and thermodynamics of drug binding. Nat. Commun..

[10] Boehr D.D., Nussinov R., Wright P.E. (2009). The role of dynamic conformational ensembles in biomolecular recognition. Nat. Chem. Biol..

[11] Boura E., Rózycki B., Herrick D.Z., Chung H.S., Vecer J., Eaton W.A., Cafiso D.S., Hummer G., Hurley J.H. (2011). Solution structure of the ESCRT-I complex by small-angle X-ray scattering, EPR, and FRET spectroscopy. Proc. Natl. Acad. Sci. USA.

[12] Ward A.B., Sali A., Wilson I.A. (2013). Integrative structural biology. Science.

[13] Lu X., Chen J., Huang J. (2025). The continuous evolution of biomolecular force fields. Structure.

[14] Bonomi M., Heller G.T., Camilloni C., Vendruscolo M. (2017). Principles of protein structural ensemble determination. Curr. Opin. Struct. Biol..

[15] Gomes G.N.W., Krzeminski M., Namini A., Martin E.W., Mittag T., Head-Gordon T., Forman-Kay J.D., Gradinaru C.C. (2020). Conformational ensembles of an intrinsically disordered protein consistent with NMR, SAXS, and single-molecule FRET. J. Am. Chem. Soc..

[16] Yu L., Brüschweiler R. (2022). Quantitative prediction of ensemble dynamics, shapes and contact propensities of intrinsically disordered proteins. PLoS Comput. Biol..

[17] Mittermaier A., Kay L.E. (2006). New tools provide new insights in NMR studies of protein dynamics. Science.

[18] Salmon L., Nodet G., Ozenne V., Yin G., Jensen M.R., Zweckstetter M., Blackledge M. (2010). NMR Characterization of long-range order in intrinsically disordered proteins. J. Am. Chem. Soc..

[19] Konrat R. (2014). NMR contributions to structural dynamics studies of intrinsically disordered proteins. J. Magn. Reson..

[20] Adamski W., Salvi N., Maurin D., Magnat J., Milles S., Jensen M.R., Abyzov A., Moreau C.J., Blackledge M.A. (2019). A unified description of intrinsically disordered protein dynamics under physiological conditions using NMR spectroscopy. J. Am. Chem. Soc..

[21] Konarev P.V., Volkov V.V., Sokolova A.V., Koch M.H.J., Svergun D.I. (2003). PRIMUS: A Windows PC-based system for small-angle scattering data analysis. J. Appl. Cryst..

[22] Aznauryan M., Delgado L., Soranno A., Nettels D., Huang J., Labhardt A.M., Grzesiek S., Schuler B. (2016). Comprehensive structural and dynamical view of an unfolded protein from the combination of single-molecule FRET, NMR, and SAXS. Proc. Natl. Acad. Sci. USA.

[23] Schuler B. (2013). Single-molecule FRET of protein structure and dynamics—A primer. J. Nanobiotechnol..

[24] Bonomi M., Vendruscolo M. (2019). Determination of protein structural ensembles using cryo-electron microscopy. Curr. Opin. Struct. Biol..

[25] Fraser J.S., van den Bedem H., Samelson A.J., Lang P.T., Holton J.M., Echols N., Alber T. (2011). Accessing protein conformational ensembles using room-temperature X-ray crystallography. Proc. Natl. Acad. Sci. USA.

[26] Rappsilber J. (2011). The beginning of a beautiful friendship: Cross-linking/mass spectrometry and modelling of proteins and multi-protein complexes. J. Struct. Biol..

[27] Leitner A., Joachimiak L.A., Unverdorben P., Walzthoeni T., Frydman J., Förster F., Aebersold R. (2014). Chemical cross-linking/mass spectrometry targeting acidic residues in proteins and protein complexes. Proc. Natl. Acad. Sci. USA.

[28] Piersimoni L., Kastritis P.L., Arlt C., Sinz A. (2022). Cross-linking mass spectrometry for investigating protein conformations and protein-protein interactions—A method for all seasons. Chem. Rev..

[29] Huang Y.J., Brock K.P., Ishida Y., Swapna G.V., Inouye M., Marks D.S., Sander C., Montelione G.T., Wand A.J. (2019). Chapter Thirteen—Combining Evolutionary Covariance and NMR Data for Protein Structure Determination. Biological NMR Part A.

[30] Orioli S., Larsen A.H., Bottaro S., Lindorff-Larsen K. (2020). How to learn from inconsistencies: Integrating molecular simulations with experimental data. Prog. Mol. Biol. Transl. Sci..

[31] Shaw D.E., Deneroff M.M., Dror R.O., Kuskin J.S., Larson R.H., Salmon J.K., Young C., Batson B., Bowers K.J., Chao J.C. (2008). Anton, a special-purpose machine for molecular dynamics simulation. Commun. ACM.

[32] Tozzini V. (2010). Minimalist models for proteins: A comparative analysis. Q. Rev. Biophys..

[33] Kmiecik S., Gront D., Kolinski M., Wieteska L., Dawid A.E., Kolinski A. (2016). Coarse-grained protein models and their applications. Chem. Rev..

[34] Noid W.G. (2023). Perspective: Advances, challenges, and insight for predictive coarse-grained models. J. Phys. Chem. B.

[35] Borges-Araujo L., Patmanidis I., Singh A.P., Santos L.H.S., Sieradzan A.K., Vanni S., Czaplewski C., Pantano S., Shinoda W., Monticelli L. (2023). Pragmatic coarse-graining of proteins: Models and applications. J. Chem. Theory Comput..

[36] Berman H.M., Westbrook J., Feng Z., Gilliland G., Bhat T.N., Weissig H., Shindyalov I.N., Bourne P.E. (2000). The Protein Data Bank. Nucleic Acids Res..

[37] Liwo A., Sieradzan A.K., Lipska A.G., Czaplewski C., Joung I., Żmudzińska W., Hałabis A., Ołdziej S. (2019). A general method for the derivation of the functional forms of the effective energy terms in coarse-grained energy functions of polymers. III. Determination of scale-consistent backbone-local and correlation potentials in the UNRES force field and force-field calibration and validation. J. Chem. Phys..

[38] Sieradzan A.K., Czaplewski C., Krupa P., Mozolewska M.A., Karczyńska A.S., Lipska A.G., Lubecka E.A., Gołaś E., Wirecki T., Makowski M., Muñoz V. (2022). Modeling the structure, dynamics, and transformations of proteins with the UNRES force field. Protein Folding: Methods and Protocols.

[39] Sieradzan A.K., Sans-Dueño J., Lubecka E.A., Czaplewski C., Lipska A.G., Leszczyński H., Ocetkiewicz K.M., Proficz J., Czarnul P., Krawczyk H. (2023). Optimization of parallel implementation of UNRES package for coarse-grained simulations to treat large proteins. J. Comput. Chem..

[40] Wüthrich K. (1986). NMR of Proteins and Nucleic Acids.

[41] Schwieters C.D., Kuszewski J., Clore G.M. (2006). Using Xplor-NIH for NMR molecular structure determination. Prog. Nucl. Magn. Reson. Spectrosc..

[42] Torda A.E., Scheek R.M., van Gunsteren W.F. (1989). Time-dependent distance restraints in molecular dynamics simulations. Chem. Phys. Lett..

[43] Torda A.E., Brunne R.M., Huber T., Kessler H., van Gunsteren W.F. (1993). Structure refinement using time-averaged J-coupling constant restraints. J. Biomol. NMR.

[44] Bonvin A.M.J.J., Boelens R., Kaptein R. (1994). Time- and ensemble-averaged direct NOE restraints. J. Biomol. NMR.

[45] Hansen N., Heller F., Schmid N., van Gunsteren W.F. (2014). Time-averaged order parameter restraints in molecular dynamics simulations. J. Biomol. NMR.

[46] Pearlman D.A., Case D.A., Caldwell J.W., Ross W.S., Cheatham T.E., DeBolt S., Ferguson D., Seibel G., Kollman P. (1995). AMBER: A package of computer programs for applying molecular mechanics, normal mode analysis, molecular dynamics and free energy calculations to simulate the structural and energetic properties of molecules. Comput. Phys. Commun..

[47] Co N.T., Czaplewski C., Lubecka E.A., Liwo A. (2025). Implementation of time-averaged restraints with UNRES coarse-grained model of polypeptide chains. J. Chem. Theory Comput..

[48] Lubecka E.A., Liwo A. (2021). ESCASA: Analytical estimation of atomic coordinates from coarse-grained geometry for nuclear-magnetic-resonance-assisted protein structure modeling. I. Backbone and H*β* protons. J. Comput. Chem..

[49] Nikiforovich G.V., Vesterman B., Betins J., Podins L. (1987). The space structure of a conformationally labile oligopeptide in solution: Angiotensin. J. Biomol. Struct. Dyn..

[50] Bonomi M., Camilloni C., Cavalli A., Vendruscolo M. (2016). Metainference: A Bayesian inference method for heterogeneous systems. Sci. Adv..

[51] Schwieters C.D., Bermejo G.A., Clore G.M. (2018). Xplor-NIH for molecular structure determination from NMR and other data sources. Protein Sci..

[52] Medeiros Selegato D., Bracco C., Giannelli C., Parigi G., Luchinat C., Sgheri L., Ravera E. (2020). Comparison of different reweighting approaches for the calculation of conformational variability of macromolecules from molecular simulations. ChemPhysChem.

[53] Pitera J.W., Chodera J.D. (2012). On the use of experimental observations to bias simulated ensembles. J. Chem. Theory Comput..

[54] Roux B., Weare J. (2013). On the statistical equivalence of restrained-ensemble simulations with the maximum entropy method. J. Chem. Phys..

[55] Hummer G., Köfinger J. (2015). Bayesian ensemble refinement by replica simulations and reweighting. J. Chem. Phys..

[56] Olsson S., Cavalli A. (2015). Quantification of entropy-loss in replica-averaged modeling. J. Chem. Theory Comput..

[57] Cavalli A., Camilloni C., Vendruscolo M. (2013). Molecular dynamics simulations with replica-averaged structural restraints generate structural ensembles according to the maximum entropy principle. J. Chem. Phys..

[58] Camilloni C., Cavalli A., Vendruscolo M. (2013). Replica-averaged metadynamics. J. Chem. Theory Comput..

[59] Camilloni C., Vendruscolo M. (2014). Statistical mechanics of the denatured state of a protein using replica-averaged metadynamics. J. Am. Chem. Soc..

[60] Raddi R.M., Marshall T., Ge Y., Voelz V.A. (2025). Model selection using replica averaging with Bayesian inference of conformational populations. J. Chem. Theory Comput..

[61] Birkhoff G.D. (1931). Proof of the ergodic theorem. Proc. Natl. Acad. Sci. USA.

[62] von Neumann J.V. (1932). Proof of the quasi-ergodic hypothesis. Proc. Natl. Acad. Sci. USA.

[63] Rhee Y.M., Pande V.S. (2003). Multiplexed-replica exchange molecular dynamics method for protein folding simulation. Biophys. J..

[64] Czaplewski C., Kalinowski S., Liwo A., Scheraga H.A. (2009). Application of multiplexed replica exchange molecular dynamics to the UNRES force field: Tests with *α* and *α*+*β* proteins. J. Chem. Theory Comput..

[65] Schrödinger, LLC (2025). The PyMOL Molecular Graphics System.

[66] Williams T., Kelley C., Bröker H.-B., Campbell J., Cunningham R., Denholm D., Elber G., Fearick R., Grammes C., Hart L. (2025). *Gnuplot: An Interactive Plotting Program*, Version 6.0.3. https://gnuplot.sourceforge.net/.

[67] Köfinger J., Hummer G. (2024). Encoding prior knowledge in ensemble refinement. J. Chem. Phys..

[68] Kelley D., Galbraith P. (2023). *GRI: Scientific Graphics Language*, Version 2.12.23. http://gri.sourceforge.net/.

[69] Lorieau J.L., Louis J.M., Schwieters C.D., Bax A. (2012). pH-triggered, activated-state conformations of the influenza hemagglutinin fusion peptide revealed by NMR. Proc. Natl. Acad. Sci. USA.

[70] Güntert P., Buchner L. (2015). Combined automated NOE assignment and structure calculation with CYANA. J. Biomol. NMR.

[71] Zaborowski B., Jagieła D., Czaplewski C., Hałabis A., Lewandowska A., Żmudzińska W., Ołdziej S., Karczyńska A., Omieczynski C., Wirecki T. (2015). A maximum-likelihood approach to force-field calibration. J. Chem. Inf. Model..

[72] Sieradzan A.K., Makowski M., Augustynowicz A., Liwo A. (2017). A general method for the derivation of the functional forms of the effective energy terms in coarse-grained energy functions of polymers. I. Backbone potentials of coarse-grained polypeptide chains. J. Chem. Phys..

[73] Liwo A., Khalili M., Czaplewski C., Kalinowski S., Ołdziej S., Wachucik K., Scheraga H.A. (2007). Modification and optimization of the United-Residue (UNRES) potential energy function for canonical simulations. I. Temperature dependence of the effective energy function and tests of the optimization method with single training proteins. J. Phys. Chem. B.

[74] Khalili M., Liwo A., Rakowski F., Grochowski P., Scheraga H.A. (2005). Molecular dynamics with the united-residue model of polypeptide chains. I. Lagrange equations of motion and tests of numerical stability in the microcanonical mode. J. Phys. Chem. B.

[75] Khalili M., Liwo A., Jagielska A., Scheraga H.A. (2005). Molecular dynamics with the united-residue model of polypeptide chains. II. Langevin and Berendsen-bath dynamics and tests on model *α*-helical systems. J. Phys. Chem. B.

[76] Swope W.C., Andersen H.C., Berens P.H., Wilson K.R. (1982). A computer simulation method for the calculation of equilibrium constants for the formation of physical clusters of molecules: Application to small water clusters. J. Chem. Phys..

[77] Hansmann U.H.E. (1997). Parallel tempering algorithm for conformational studies of biological molecules. Chem. Phys. Lett..

[78] Trebst S., Troyer M., Hansmann U.H.E. (2006). Optimized parallel tempering simulations of proteins. J. Chem. Phys..

[79] Kumar S., Rosenberg J.M., Bouzida D., Swendsen R.H., Kollman P.A. (1992). The weighted histogram analysis method for free-energy calculations on biomolecules. I. The method. J. Comput. Chem..

[80] Nishikawa K., Momany F.A., Scheraga H.A. (1974). Low-energy structures of two dipeptides and their relationship to bend conformations. Macromolecules.

[81] Lubecka E.A., Liwo A. (2022). A coarse-grained approach to NMR-data-assisted modeling of protein structures. J. Comput. Chem..

[82] Lange O.F., Rossi P., Sgourakis N.G., Song Y., Lee H.W., Aramini J.M., Ertekin A., Xiao R., Acton T.B., Montelione G.T. (2012). Determination of solution structures of proteins up to 40 kDa using CS-Rosetta with sparse NMR data from deuterated samples. Proc. Natl. Acad. Sci. USA.

[83] Everett J.K., Tejero R., Murthy S.B.K., Acton T.B., Aramini J.M., Baran M.C., Benach J., Cort J.R., Eletsky A., Forouhar F. (2016). A community resource of experimental data for NMR / X-Ray crystal structure pairs. Protein Sci..

[84] Lee J., Liwo A., Scheraga H.A. (1999). Energy-based de novo protein folding by conformational space annealing and an off-lattice united-residue force field: Application to the 10–55 fragment of staphylococcal protein A and to apo calbindin D9K. Proc. Natl. Acad. Sci. USA.

[85] Heo L., Feig M. (2023). One Bead Per Residue Can Describe All-Atom Protein Structures. cg2all Version v1.3.1. https://github.com/huhlim/cg2all.

[86] Heo L., Feig M. (2024). One bead per residue can describe all-atom protein structures. Structure.

[87] Murtagh F., Heck A. (1987). Multivariate Data Analysis.

[88] Gorba C., Miyashita O., Tama F. (2008). Normal-mode flexible fitting of high-resolution structure of biological molecules toward one-dimensional low-resolution data. Biophys. J..

[89] Yang S., Roux B. (2011). EROS: Better than SAXS!. Structure.

[90] Kimanius D., Pettersson I., Schluckebier G., Lindahl E., Andersson M. (2015). SAXS-guided metadynamics. J. Chem. Theory Comput..

[91] Leśniewski M., Pyrka M., Czaplewski C., Co N.T., Jiang Y., Gong Z., Tang C., Liwo A. (2024). Assessment of two restraint potentials for coarse-grained chemical-cross-link-assisted modeling of protein structures. J. Chem. Inf. Model..

