# Supplementary material for: Implementation of Replica-Averaged Restraints from Nuclear Magnetic Resonance Measurement with UNRES Coarse Grained Model of Polypeptide Chains
Source: Molecules. 2025 Nov 10;30(22):4354. doi: 10.3390/molecules30224354 (PMC12654794; doi:10.3390/molecules30224354)
Supplement: Supplementary file 1 [file molecules-30-04354-s001.zip › SupplTables.pdf]

Supplementary Material (Tables)

Implementation of Replica-Averaged Restraints from Nuclear Magnetic Resonance Measurement with UNRES Coarse Grained Model of Polypeptide Chains

Leonid Shirkov, Cezary Czaplewski and Adam Liwo*

Table S1: Right RMSDs from the upper distance boundaries (ρ_u^+s), the total numbers of violated restraints (n_{viol}), and the numbers of restraints violated by more than 2 Å (N_{viol}) for the 2LWA, 1PQX, 2KW5, and 2KZN proteins from MREMD simulations with 12 quadruplexed replicas. The average distances were calculated from conformational ensembles at T = 280 K.

Protein	Calc. mode	whole ensemble[a]			20 structures[b]			PDB[c]		
		ρ_u^+ [Å]	n_{viol}	N_{viol}	ρ_u^+ [Å]	n_{viol}	N_{viol}	ρ_u^+ [Å]	n_{viol}	N_{viol}
2LWA	no ave.	0.41	30	2	0.65	41	4	0.44	41	1
	rep. ave.	0.10	18	0	0.24	22	0			
	time ave.[d]	0.10	17	0	0.28	30	1			
2KW5	no ave.	0.40	227	5	0.58	283	17	0.66	423	21
	rep. ave.	0.18	121	0	0.29	199	0			
	time ave.[d]	0.25	166	0	0.44	286	4			
2KZN	no ave.	0.51	149	7	0.68	228	13	0.61	227	9
	rep. ave.	0.20	102	0	0.36	158	1			
	time ave.[d]	0.31	153	2	0.60	204	11			
1PQX	no ave.	0.11	33	0	0.20	51	3	0.02	6	0
	rep. ave.	0.03	12	0	0.10	37	1			
	time ave.[d]	0.07	20	0	0.26	53	4			

[a]Calculated from the structure converted to the all-atom representation.

[b]Calculated from the representatives of 20 families after minimum-variance dissection of the respective ensemble and converting to the all-atom representation.

[c]Calculated from the respective PDB ensembles in Co et al., *J. Chem. Theory Comput.*, **21**, 1476-1493 (2025).

[d]Time averaging with the memory window of τ = 489 ps and full average update every 1000 steps, data from Co et al., *J. Chem. Theory Comput.*, **21**, 1476-1493 (2025).

Table S2: Right RMSDs from the upper distance boundaries (ρ_u^+s),the total numbers of violated restraints (n_{viol}), and the numbers of restraints violated by more than 2 Å (N_{viol}) for the 2LWA, 1PQX, 2KW5, and 2KZN proteins calculated from the ESCASA-estimated proton coordinates from four schemes of NMR-data-restrained MREMD simulations with UNRES in the replica-average mode and without averaging. The average distances were calculated from conformational ensembles at $T = 280$ K.

Protein	Replica scheme[a]											
	24×2			12×4			8×6			6×8		
	ρ_u^+ [Å]	n_{viol}	N_{viol}	ρ_u^+ [Å]	n_{viol}	N_{viol}	ρ_u^+ [Å]	n_{viol}	N_{viol}	ρ_u^+ [Å]	n_{viol}	N_{viol}
Replica averaged												
2LWA	0.25	28	0	0.18	23	0	0.19	27	0	0.20	24	0
2KW5	0.29	203	2	0.30	128	4	0.34	117	7	0.38	116	7
2KZN	0.27	138	0	0.27	78	1	0.30	62	2	0.32	72	2
1PQX	0.02	18	0	0.01	7	0	0.01	4	0	0.01	7	0
No average												
2LWA	0.49	50	3	0.49	50	3	0.49	49	3	0.49	48	3
2KW5	0.48	314	7	0.49	332	8	0.48	314	7	0.51	325	13
2KZN	0.47	184	7	0.47	183	6	0.54	187	12	0.54	187	12
1PQX	0.06	30	0	0.06	33	0	0.06	36	0	0.06	36	0

[a]In the $N_T \times M$ notation, N_T is the number of replicas and M is the number of multiplexings per replica.
